# Transparency in quality of radiotherapy for breast cancer in the Netherlands: a national registration of radiotherapy-parameters

**DOI:** 10.1186/s13014-022-02043-0

**Published:** 2022-04-12

**Authors:** Nansi Maliko, Marcel R. Stam, Liesbeth J. Boersma, Marie-Jeanne T. F. D. Vrancken Peeters, Michel W. J. M. Wouters, Eline KleinJan, Maurice Mulder, Marion Essers, Coen W. Hurkmans, Nina Bijker

**Affiliations:** 1 Scientific Bureau, Dutch Institute for Clinical Auditing, Leiden, The Netherlands; 2 Department of Surgical Oncology, Netherlands Cancer Institute/Antoni Van Leeuwenhoek, Amsterdam, The Netherlands; 3 Radiotherapiegroep, Arnhem, The Netherlands; 4 Department of Radiation Oncology (Maastro), GROW School for Oncology, Maastricht University Medical Centre+, Maastricht, The Netherlands; 5 Department of Surgery, AmsterdamUMC, Amsterdam, the Netherlands; 6 Department of Biomedical Data Sciences, Leiden University Medical Centre, Leiden, The Netherlands; 7 Trusted Third Party, Medical Research Data Management, Deventer, The Netherlands; 8 Institute Verbeeten, Tilburg, The Netherlands; 9 Department of Radiation Oncology, Catharina Hospital, Eindhoven, The Netherlands; 10 Department of Radiation Oncology, AmsterdamUMC, Meibergdreef 9, 1105 AZ Amsterdam, The Netherlands

**Keywords:** Radiotherapy, Breast cancer, National clinical audit, NABON Breast Cancer Audit, Quality of care

## Abstract

**Background:**

Radiotherapy (RT) is part of the curative treatment of approximately 70% of breast cancer (BC) patients. Wide practice variation has been reported in RT dose, fractionation and its treatment planning for BC. To decrease this practice variation, it is essential to first gain insight into the current variation in RT treatment between institutes. This paper describes the development of the NABON Breast Cancer Audit-Radiotherapy (NBCA-R), a structural nationwide registry of BC RT data of all BC patients treated with at least surgery and RT.

**Methods:**

A working group consisting of representatives of the BC Platform of the Dutch Radiotherapy Society selected a set of dose volume parameters deemed to be surrogate outcome parameters, both for tumour control and toxicity. Two pilot studies were carried out in six RT institutes. In the first pilot study, data were manually entered into a secured web-based system. In the second pilot study, an automatic Digital Imaging and Communications in Medicine (DICOM) RT upload module was created and tested.

**Results:**

The NBCA-R dataset was created by selecting RT parameters describing given dose, target volumes, coverage and homogeneity, and dose to organs at risk (OAR). Entering the data was made mandatory for all Dutch RT departments. In the first pilot study (N = 1093), quite some variation was already detected. Application of partial breast irradiation varied from 0 to 17% between the 6 institutes and boost to the tumour bed from 26.5 to 70.2%. For patients treated to the left breast or chest wall only, the average mean heart dose (MHD) varied from 0.80 to 1.82 Gy; for patients treated to the breast/chest wall only, the average mean lung dose (MLD) varied from 2.06 to 3.3 Gy. In the second pilot study 6 departments implemented the DICOM-RT upload module in daily practice. Anonymised data will be available for researchers via a FAIR (Findable, Accessible, Interoperable, Reusable) framework.

**Conclusions:**

We have developed a set of RT parameters and implemented registration for all Dutch BC patients. With the use of an automated upload module registration burden will be minimized. Based on the data in the NBCA-R analyses of the practice variation will be done, with the ultimate aim to improve quality of BC RT.

*Trial registration* Retrospectively registered.

**Supplementary Information:**

The online version contains supplementary material available at 10.1186/s13014-022-02043-0.

## Background

Radiotherapy (RT) is part of the treatment in approximately 70% of breast cancer (BC) patients, with 97.3% after breast conserving surgery (BCS) and 26.1% after mastectomy [1, 2]. Despite of the presence of national guidelines on RT in the Netherlands, previous studies have shown that there is still variation in the use of RT, e.g., a wide variation in the use of boost irradiation in patients that underwent BCS [3]. Further, a survey done in 2013 by the BC Platform of the Dutch Society for RT and Oncology (NVRO), showed significant variation in breast RT treatment planning between all 19 RT institutes in the Netherlands. Examples included the definition of target volumes, treatment planning margins and applied radiation technique (Volumetric-Modulated Arc Therapy (VMAT), Intensity Modulated Radiotherapy (IMRT) vs 3D conformal radiotherapy (3DCRT)).

In breast cancer RT this variation is due to the fact that, apart from available technology and differences in delineation practices and prescribed dose schedules, physicians make different choices when deciding what is an optimal RT plan. For example, some prefer optimal sparing of the heart, even at the cost of underdosage of the target volume. Or, when giving a boost, some would accept a slightly higher heart dose to prevent a more non-conformal plan at the cost of a high boost volume in the breast. Furthermore, patient and tumour-related factors are important in the decision-making regarding one RT plan versus the other [4].

The observations described above have increased the wish to gain more insight into the variation in RT on patient level, with the ultimate aim to improve RT quality. Several papers have been published describing national initiatives to add detailed RT data to national registries. However some have narrowed their scope to study cohorts only, while the others have not reported definite implementation nor preliminary outcomes [5–8]. In the Netherlands, quality of BC care is structurally being measured for all surgically treated BC patients, by a multidisciplinary set of quality indicators (QIs) in the NABON (“National Breast Cancer Organization Netherlands”) Breast Cancer Audit (NBCA). The NBCA multidisciplinary registration contains tumour and treatment characteristics of all surgically treated BC patients, facilitated by the Dutch Institute for Clinical Auditing (DICA) [9, 10]. Earlier research has shown that the NBCA QIs allow nationwide comparison of BC care between hospitals and reduction of practice variation by annual-cycles benchmark feedback [9]. Through clinical auditing it is thus possible to improve quality of medical care and patient outcomes [11]. For RT however, only limited RT data from the individual patient were recorded in the NBCA (i.e. whether or not RT was given, with/without boost to the tumour bed and whether local/locoregional RT was given). Therefore, the NVRO started the NBCA Radiotherapy (NBCA-R) project. The main aim of this project was to establish a robust set of RT parameters, reflecting the quality of RT provided to BC patients. To facilitate widespread acceptance of the registration of these parameters, registration burden had to be minimalised. Therefore, the project required the development of an automatic upload system for these parameters. The aim of this paper is to describe the process of the development of the NBCA-R, a Dutch nationwide mandatory RT-registry for BC patients. The NBCA-R will provide data of all BC patients treated with at least surgery and radiotherapy and real-world data for better understanding the daily clinical practice. To demonstrate the feasibility of the NBCA-R, the results of the first pilot studies (validation of the data dictionary and development of an automated Digital Imaging and Communications in Medicine (DICOM) RT upload system) will be discussed.

## Methods

### Development of the dataset

First, a working group (WG) was composed of representatives of the BC Platform of the NVRO. This WG developed a set of RT parameters deemed to be representative for outcome, both for tumour control and toxicity.

In order to be able to compare to other international datasets, the plan description criteria from the international commission on radiation units (ICRU) and measurements criteria were followed to evaluate the dose to the target volume [12]. Only a few parameters were changed or added. For organs at risk (OAR) consensus was reached on the relevant dose-volume parameters based on literature [13–20]. National consensus was reached for the complete set, which was approved at an NVRO national meeting. A data dictionary for the complete registry is published on the DICA website (Table 1).

**Table 1:** Overview of the approved NBCA-R set

Section	Variables
Identification	
	Sex
	Date of birth
General	
	RT institute
	Date planning CT scan
	Laterality tumour
Total treatment time	
	Date first fraction RT
	Date last fraction RT
	Date of the first RT consultation of this radiation plan
Target areas	
	Whole breast: yes/no
	Partial breast: yes/no
	Chest wall: yes/no
	Boost breast or chest wall: yes/no
	Interpectoral lymph nodes: yes/no
	Axillary lymph node levels I-II: yes/no
	Axillary lymph node levels III-IV: yes/no
	Internal mammary lymph nodes: yes/no
	Boost lymph nodes: yes/no
Prescribed dose	
Elective dose (local/regional)	Number of fractions
Elective dose (local/regional)	Dose per fraction (Gy)^a^
Dose on PTV boost tumour bed	Number of fractions
Dose on PTV boost tumour bed	Dose per fraction (Gy)
Dose on PTV boost lymph nodes	Number of fractions
Dose on PTV boost lymph nodes	Dose per fraction (Gy)
Type of boost	Simultaneous integrated boost vs sequential boost
Doses distribution on target areas	
PTV-elective^b^	D2% (Gy)
PTV-elective^b^	D98% (Gy)
PTV-elective^b^	Dmean (Gy)
PTV-Boost-tumour bed	V95% of prescribed dose (%)
PTV-Boost-tumour bed	D2% (Gy)
PTV-Boost-tumour bed	D98% (Gy)
PTV-Boost-tumour bed	Dmean (Gy)
PTV-Boost- axillary lymph nodes	V95% of prescribed dose (%)
PTV-Boost- axillary lymph nodes	D2% (Gy)
PTV-Boost- axillary lymph nodes	D98% (Gy)
PTV-Boost- axillary lymph nodes	Dmean (Gy)
PTV-elective minus PTV boost^c^	D2% (Gy)
PTV-elective minus PTV boost^c^	D98% (Gy)
PTV-elective minus PTV boost^c^	Dmean (Gy)
Dose in normal tissues	
Heart	D2% (Gy)
Heart	Dmean (Gy)
Ipsilateral breast	V95% of the prescribed boost dose in the body (mL)
	Volume of CTV breast (mL)
Lungs	Mean dose (Gy), both lungs

Gy, Gray; CT-scan, computed tomography scan; RT, radiotherapy; mL, milliliter; PTV, planning target volume

^a^Prescribed dose to the normalisation point

^b^All PTVs together: PTV breast/chest wall, PTV axillary lymph nodes including PTV boost if given

^c^All PTVs together: PTV breast/chest wall, PTV axillary lymph nodes minus PTV boost if given

Information about patient characteristics (age and performance status), tumour characteristics (tumour stage and biology), process times (‘time between surgery and RT’ and ‘time between adjuvant systemic therapy and RT’), and clinical disease management (surgery, systemic therapy) were not part of the NBCA-R dataset, since these data are already collected in the NBCA and could be obtained through linkage between databases.

The inclusion criteria of the NBCA-R were chosen to be identical to the NBCA, i.e. all surgically treated patients with primary invasive BC or ductal carcinoma in situ (DCIS), who have also received RT. Patients diagnosed with lobular carcinoma in situ, phyllodes tumours, sarcomas and lymphomas were excluded.

### Trusted third party and software development

A web-based portal was developed for manual entering of data. In addition, an automated upload system was developed, to extract data directly from the RT planning data. Subsequently, two pilot studies were performed: (1) manual entrance of the RT parameters, to investigate the feasibility and validity of the dataset; (2) entrance via an automated upload, to investigate the feasibility of the developed automated upload module.

To comply with the European General Data Protection Regulation (GDPR) all data were entered on servers from and processed by a trusted third party, Medical Research Data Management (MRDM). This company offers connectivity services, database storage and verification services and anonymizes data before there are sent to the DICA database [21]. MRDM also developed the software to extract all data from the RT planning data. In order to comply with the GDPR all testing department had to have data processor agreements with MRDM.

### NBCA-R pilot studies

In the first pilot study data were manually entered into a secured web-based system, by data-managers, RT technicians, RT physicists or radiation oncologists.

After the first pilot study the participants were asked to give their opinion about the usability of the system, availability of the parameters in clinical data systems and time needed to register data per patient. Based on the feedback, the set of parameters was adjusted and definitions were fine-tuned.

To reduce registration burden, the NBCA-R project also required the development of an ‘automatic’ extraction of RT data from the raw RT treatment planning-data (DICOM-RT). In the second pilot study this automated upload system was tested and implemented in daily practice.

### Funding

The creation of NBCA-R was funded by a grant from Quality foundation of the Dutch Federation of Medical Specialists (SKMS) [22]. SKMS provides grants exclusively to national societies of medical specialists for projects related to improvement of quality of care. Since 2020, the cost of NBCA-R, including the data registration and automatic upload is completely covered by an umbrella organization of ten healthcare insurance companies in the Netherlands (ZN) [23]. A startup fee of €2000,- per registry needs to be paid by the hospitals. ZN also funds all other DICA registries, but does not influence its workings [24].

## Results

### Definite set of radiotherapy parameters

The NBCA-R dataset had to include some general patient information, e.g., postal code, date of birth and gender. The WG concluded that parameters indicative for quality of BC RT should preferably consist of outcome parameters, like (loco-regional-) tumour control, toxicity, and patient reported outcomes measures (PROMs). However, the effect of RT on local recurrence and toxicity can only be measured years after treatment, which would hamper short-cycled adaptation and improvement of quality in case of deviation from quality indicators. Therefore, the group aimed for registering parameters expected to be related to these long-term outcome parameters.

Five main categories of RT-specific parameters were considered essential: (1) which target volumes were irradiated, (2) dose and fractionation schedule, (3) dose-volume parameters with respect to target volume coverage and dose homogeneity, (4) dose-volume parameters of OARs, (5) and a limited number of patient characteristics, such as the volume of the Clinical Target Volume (CTV) of the breast as a surrogate for breast size (Table 1). For the target volumes the following sub-volumes were defined: breast, tumour bed, chest wall, axilla levels I–II, interpectoral, axilla levels III–IV, and internal mammary lymph nodes. Since delineation of target volumes can heavily affect dose-volume histogram (DVH) parameters, consensus was reached that target volumes had to be delineated according to the ESTRO atlas [25]. For each target volume, the prescribed number of fractions and dose per fraction had to be recorded.

Dose-volume parameters were largely selected based on the ICRU 83 criteria, i.e. to evaluate coverage and dose-homogeneity of the Planning Target Volume (PTV), the D98% (i.e. the dose given to at least 98% of the PTV) and the D2% (i.e. highest dose given to 2% of the PTV, i.e. the near maximum dose) were selected, as well as the Dmean [12]. Dmean was preferred above Dmedian (recommended by ICRU) since all departments (and treatment planning systems (TPS)) use this parameter in daily practice. These parameters were required for the total PTV including the regional subPTVs, and for the boost PTV. To evaluate the dose received by the OAR, the Dmean was selected for both heart and lungs, and D2% for the heart. Dmean for the heart was chosen since it was found to be related to the risk on developing an acute coronary event [13]; Dmean for the lungs was chosen since it has been shown to be related to the risk of developing lung cancer [26] and radiation pneumonitis [27]. The V95% of the body (volume of the body receiving at least 95% of the prescribed boost dose) was selected in case of boost irradiation, as a measure to quantify the high dose volume, which has been reported to be related to cosmetic outcome [28, 29].

To allow linkage of the NBCA-R with the general NBCA-dataset, social security number could not be used due to privacy regulations. Therefore, some process parameters were added to the NBCA-R dataset, that were also included in the NBCA dataset: date of planning computed tomography scan (CT-scan), date of the first consultation with the radiation oncologist, start and end date RT (Table 1).

### Pilot study 1: manual data-entry

Six radiotherapy institutes participated in the first pilot study, and entered data of a total of 1093 (range: 53–404) patients with a mean age of 61.3 years (range: 25 years–98 years). It took about 10–15 min to manually enter the RT data of each individual patient. For an average Dutch RT institute treating 500 BC patients per year, manual registration would take over 83 h. The pilot study revealed the feasibility of registering the required data in the database, since there were little or no missing data. After the pilot phase some adjustments had to be made to the database for improvement of some definitions and datapoints. In the initial phase, PTV-elective (all PTVs together minus the PTV-boost) was requested in the survey. Since several RT institutes did not create this Region of Interest in daily practice, this parameter was made optional. An additional parameter PTV-elective total (all PTVs including also PTV boost) was added to the dataset. In the first dataset only the parameter “PTV boost” was defined. As a result of the second pilot study separate PTVs for a breast-boost and lymph node-boost were added.

**Fig. 1:**
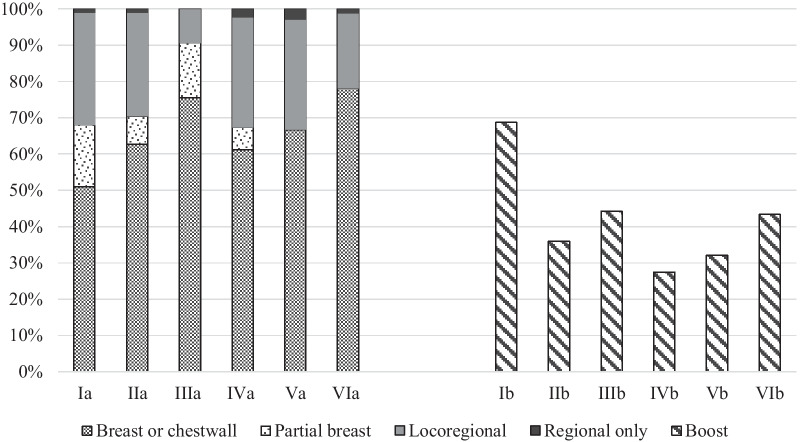
Percentage of BC patients that received radiotherapy on different target volumes for the six pilot institutes (I–VI). **a** Elective target volumes. **b** Percentage of patients who received a boost on the tumour bed, patients who receive partial breast radiation (PBI) were excluded

*Observed variation in registered RT parameters:* In this pilot study 692 patients (63.3%) received RT solely to the breast or chest wall (range 51.0%-78.1%). Partial breast radiation was given to 7.1% of the patients, with quite some variation between the radiotherapy institutes (range 0%-17.0%). 27.8% of the patients received locoregional RT (range 9.4–31.0%), with 1.7% receiving nodal RT without local RT (range 0–2.9%) (Fig. 1).

306 of patients treated with BCS (36.6%) received a boost to the tumour bed, with a wide variation between the institutes (range 27.4–68.7%) (Fig. 1). The most frequently applied (97.1%) fractionation schedule to the whole breast was 15 × 2.67 Gy if no boost was delivered; in case a boost was applied, the elective volumes received 20 × 2.18 Gy, with concomitantly 20 × 2.67 Gy to the tumour bed. When PBI was applied most patients (88.5%, 69 of 78) received 15 × 2.67 Gy, 10% received 10 × 3.85 Gy (twice a day), of one patient data were missing.

Observed variation in dose-volume parameters is given in Table 2. The average coverage of the PTV (D98%) varied between the institutes from 92.0 to 95.6% of the prescribed dose, with quite some observed difference in standard deviation between the institutes. For patients treated to the left breast or chest wall only, the average mean heart dose (MHD) varied from 0.80 to 1.82 Gy; for patients treated to the breast/chest wall only, the average mean lung dose (MLD) varied from 2.06 to 3.3 Gy (Table 2).

**Table 2:** Dose volume histogram (DVH) parameters in the six pilot institutes (I–VI)

	I	II	III	IV	V	VI
Dose to PTV (homogeneity) (%)^a^						
D98% (SD)	92.0 (3.2)	94.9 (2.2)	95.2 (1.6)	96.0 (1.4)	93.4 (4.9)	95.4 (1.1)
D2% (SD)	104.0 (0.7)	104.0 (0.6)	104.0 (1.0)	103.0 (0.8)	104.0 (0.8)	104.0 (1.5)
Dmean (SD)	99.7 (0.7)	100.0 (0.4)	100.0 (0.7)	100.0 (0.9)	100.0 (0.5)	101.0 (0.6)
Dose to OAR (Gy)						
MHD (SD)^b^	0.8 (0.3)	1.7 (1.2)	1.5 (0.5)	0.9 (0.3)	1.0 (0.2)	1.8 (1.4)
D2% heart (SD)^b^	2.8 (1.1)	8.3 (6.5)	7.2 (6.3)	4.1 (3.1)	3.3 (0.9)	13.7 (14.4)
MLD (SD)^a^	2.1 (0.5)	2.9 (0.9)	2.8 (0.8)	2.9 (0.7)	2.3 (0.6)	3.3 (1.0)

SD, standard deviation, OAR, Organs at risk, MHD, mean heart dose, MLD, mean lung dose

^a^Patients who received radiotherapy to the breast only, without a boost

^b^Patients who received radiotherapy to the left breast only, without a boost

### Pilot study 2: creating an automatic upload module

The starting point for the automatic upload was the use of the standardized DICOM-RT output of all TPS in the Netherlands. The **first step** was to create software that serves as a DICOM node to receive the full DICOM-RT data (CT-scan, plan, structure and dose). At the moment an RT plan is archived in the RT institute, or sent to the Linear Accelerator from the TPS it can also easily be sent to another receiving system. In this way, providing data to the registry can be embedded in daily practice with a minimum amount of registration burden. This “DICOM node” was connected to a generic communication software package (datasafe of MRDM) that sends the data to MRDM [21]. Installation of this datasafe software package has very little hardware requirements and takes at most several hours of configuration time. The **next step** was to create software to extract the DVHs for all relevant Regions Of Interest (ROIs) and extract the relevant datapoints from these DVHs. MRDM used two open source software packages (Pydicom and Dicompyler) in order to create DVH tables and extract the specific DVH data points. These packages were embedded in an automatic script which also anonymized data and sent the data to the DICA databases. All departments were asked to compare the calculated datapoints to that from their TPS. Furthermore, MRDM validated the DVH curves using the Curve Compare software and test dataset [21, 30].

In order to identify the relevant ROIs, the institutes were required to use uniform naming of the ROIs. We chose to let the institute keep their own names instead of imposing a standard national nomenclature, to facilitate acceptance. Consequentially, a translation/mapping table had to be made for each participating institute. This table was not only used for identification of ROIs used for DVH points, but also to find out which targets had been treated: for example breast only, chest wall only, and/or lymph nodes (see Additional file 1). Tumour laterality could be determined by calculation of centre of mass of the PTV relative to the CT-scan centre.

Since the start of registration in January 2020 until April 2021, more than 5000 BC patients have been registered in 13 of the 19 radiotherapy institutes. Nine institutes registered their patients with the automatic upload module. It must be taken into account that the year 2020 has been influenced by the COVID-19 pandemic, with limited resources available to implement the registration of all BC patients treated with RT.

## Discussion

We have defined a set of relevant and valid RT parameters, the NBCA-R, that was nationally approved and accepted as an obligatory registration for quality assessment of all BC patients treated with at least surgery and RT. Subsequently an automatic upload procedure has been developed to reduce workload and to ensure sustainable implementation. The pilot studies have shown that it is very feasible to register relevant information of BC patients that received RT, both manually and with the automatic upload module, the latter with minimal registration burden. In addition, we have shown variation between the participating institutes of the first pilot study, in the given RT (e.g. target volumes, boost or no boost, and dose distribution). Although these first results are not yet adjusted for casemix, we conclude the dataset is able to find relevant variation in the given RT to BC patients.

International publications from Denmark and Sweden showed that validated RT DICOM data can be automatically registered at a national level. Nevertheless Denmark only uses the system for specific study cohorts and Sweden has not reported implementation of the system yet [5–8]. In the United Kingdom RT data, only dose and number of fractions are collected in their national dataset [31, 32]. To our knowledge this is the first study that shows that a national RT registry can be used for clinical auditing and has also been implemented in daily practice. The RT parameters make it possible to compare RT plans and plot it against clinical outcomes in order to improve the quality of RT.

With the current low locoregional recurrence rates [33, 34] the balance between treatment and side-effects is becoming increasingly important, for example resulting in de-escalation in the use of boost. Even though variation between the different institutes in the use of boost decreased over the years [3], the NBCA-R pilot data showed that this variation is still significantly visible between the different institutes. Using prospectively collected data, such as data in the NBCA-R, more insight can be gained in given doses for various indications and how variation can be reduced in the future. This has already led to new guidelines for boost RT by the BC Platform of the NVRO.

After we had defined this set of RT parameters, a new SKMS project was carried out with the aim of reaching consensus on how to define an optimal treatment plan. In that project, the relevance of the defined set of RT parameters was confirmed, and in addition consensus was obtained on (1) requirements on the values for the dose-volume parameters representing target coverage, (2) which clinical factors should be taken into account when weighing target coverage against dose to OAR [4]. The next step will be to define constraints for dose to OARs for different situations (e.g. breast only, vs breast and regional lymph nodes). The current NBCA-R set will enable evaluation of adherence, of the Dutch RT departments, to target coverage objectives guidelines, and will provide a base to define constraints for dose to OARs.

In the first pilot study, target coverage and dose homogeneity were largely within the ICRU recommendations: the mean D98% of PTV in locally treated patients was 94.5%, slightly lower than the recommended 95%. Most institutes had a mean D2% of the total doses of 104% which is well below the recommended max of 107%. However, these are only the results of six institutes. The future will have to show whether there is relevant nationwide practice variation. Homogeneity correlates with cosmetic results and patients physical complaints like pain, fibrosis and shoulder function [35–38]. However, these aspects are also influenced by many other factors, such that a case-mix correction will be required, which will be done via linking to the NBCA.

More variation was seen in the doses to the OARs. Several studies have demonstrated that patients who received irradiation have increased mortality due to ischemic heart disease [39–45]. The results of the first pilot study show that for patients who received RT to the left breast only, a difference was observed of 1 Gy between the lowest and the highest scoring institute on MHD, which according to Darby et al., could translate clinically to 7.4% increased relative risk of major coronary events [13]. Whether this also translates in a clinically relevant difference in absolute risk is dependent on the presence of cardiovascular risk factors, gender, and age [13]. Furthermore, our pilot study showed a difference of 1.24 Gy of the given MLD. Taylor et al. described an increased incidence of primary lung cancer after 10 years RT with every Gy MLD (RR, 2.10; 95%CI, 1.48–2.98; P < 0.001) [26].

The possible limitation of the first pilot study is that only a selection of institutes have participated. Also, in this pilot we have not been able to correct for casemix and the included numbers of patients are small. However, the results suggest that some variation is seen between the institutes in MHD and MLD (Table 2). Future studies based on the registration of every irradiated BC patient in the NBCA-R, and linking it to the NBCA, will allow to analyse this variation in more detail.

We realize that in a relatively small country as the Netherlands with a high level of centralization, a project like ours can be more easily performed. Nevertheless, the standardized way we used DICOM RT can be easily implemented in other countries, since every current treatment planning system is able to connect to other DICOM systems. We have shown the feasibility of an automatic upload module based on DICOM-RT data extraction integrated in daily practice. Uniform nomenclature within each institute for ROI’s was essential for the extraction of information from the DICOM-RT data. A pilot study was essential to fine-tune indicator definitions in order to be able to map them to clinically used ROI’s.

## Future perspective

Starting from January 2020, it is mandatory for all RT institutes in the Netherlands to participate in the NBCA-R registration. The first analyses from April 2021 have shown that 13 (62%) of the 19 radiotherapy institutes have been registering their BC patients (n > 5000). It is likely that the COVID-19 pandemic may have influenced this result, as such full national coverage in 2021 is expected. With all radiotherapy data present in a national database, analyses can be made on differences in irradiated target volumes and used dose-fractionation schedules. Furthermore, we could analyse variation in dose to organs at risk, such as the heart and lungs, by for example relating these doses to possible variation in the coverage of the target volume. This might form the basis of creating of and adhering to National and International guidelines of good quality radiotherapy. By merging the NBCA-R with the NBCA data it will be possible to plot the dose coverage variables against national survival data (from the Netherlands Cancer Registry). The NBCA also aims to include PROMs. As soon as these are available, DVH parameters of OARs can be correlated with PROMs, which will make it possible to correlate the effects of dose homogeneity with actual PROM like cosmetic results and patients physical complaints like pain and fibrosis. Ultimately, this will lead to the introduction of QIs in BC RT.

Furthermore, we started developing a FAIR (Findable, Accessible, Interoperable, Reusable) infrastructure for the DVH points of the NBCA-R [46, 47]. Metadata has been added to each RT parameter for description of how data is stored in the registry. To make the data findable, a unique identification number has been linked to this file, which will soon be published on fairsharing.org. The data can be requested from the scientific office of DICA and are shared upon approval of the NBCA-R scientific board (accessible). The variables with associated metadata were linked to existing Radiation Oncology Ontology (ROO) [48, 49], so that they can be unambiguously interpreted by radiation oncologists worldwide (interoperable). For this, additions will be made to the ROO set. As a result, the RT parameters become human readable and machine interpretable. The metadata contain information about the origin and acquisition of the data and is stored in databases so that the previously obtained parameters are reusable. With the emergence of several national DICOM-registrations (e.g. Sweden and Denmark [6, 7]), FAIR would make international comparisons and data exchange possible [50].

## Conclusions

We have developed a nationwide set of RT parameters that will be registered for all Dutch BC patients. An automated upload module has been developed to ensure sustainable implementation. Using these data, more insight can be gained in the quality of the given treatment, thereby enabling analyses of the practice variation, without the increase of registration burden for radiation oncologists. By combining the NBCA-R and the NBCA datasets in the future, optimal RT treatment plans can be defined and standardized to guide treatment protocols, with the ultimate aim to improve quality of BC RT.

## Supplementary Information

**Additional file 1.** Example of a mapping table to map ROI names to variable names. CT-scan, computed tomography scan; Gy, Gray; SIB, simultaneous integrated boost In order to let departments use their own ROIs names they had to fill out a mapping table. a. ROIs descriptions mentioned in the table are examples and may differ per institution b. Based on the average dose calculated in the ROIs and if within certain ranges, mapped to prescribed dose c. Based on total dose in the boost PTV (SIB: elective + boost dose; sequential: boost dose).

## Data Availability

The data that support the findings of this study are available from the Dutch Institute for Clinical Auditing (DICA) but restrictions apply to the availability of these data, which were used under license for the current study, and so are not publicly available. Data are however available from the authors upon reasonable request and with permission of DICA.

## Abbreviations

RT: Radiotherapy
BC: Breast cancer
NABON: National Breast Cancer Organization Netherlands
NBCA-R: NABON Breast Cancer Audit-Radiotherapy
NBCA: NABON Breast Cancer Audit
DICOM: Digital Imaging and Communications in Medicine
OAR: Organs at risk
MHD: Mean heart dose
MLD: Mean lung dose
FAIR: Findable, Accessible, Interoperable, Reusable
BCS: Breast conserving surgery
NVRO: Breast Cancer Platform of the Dutch Society for radiotherapy and Oncology
VMAT: Volumetric-Modulated Arc Therapy
IMRT: Intensity Modulated Radiotherapy
3DCRT: 3D conformal radiotherapy
QI: Quality indicator
DICA: Dutch Institute for Clinical Auditing
WG: Working group
ICRU: International commission on radiation units
DCIS: Ductal carcinoma in situ
GDPR: European General Data Protection Regulation
MRDM: Medical Research Data Management
SKMS: Quality foundation of the Dutch Federation of Medical Specialists
ZN: Umbrella organization of ten healthcare insurance companies in the Netherlands
PROMs: Patient reported outcomes measures
CTV: Clinical Target Volume
DVH: Dose-volume histograms
PTV: Planning Target Volume
D98%: I.e. the dose given to at least 98% of the PTV
D2%: I.e. highest dose given to 2% of the PTV, i.e. the near maximum dose
Dmean: Mean dose given
Dmedian: Median dose given
TPS: Treatment planning systems
V95% of the body: Volume of the body receiving at least 95% of the prescribed boost dose
CT-scan: Computed tomography scan
Gy: Gray
ROI: Regions Of Interest
ROO: Radiation Oncology Ontology
