# Supplementary material for: Transparency in quality of radiotherapy for breast cancer in the Netherlands: a national registration of radiotherapy-parameters
Source: Radiat Oncol. 2022 Apr 12;17:73. doi: 10.1186/s13014-022-02043-0 (PMC9003170; doi:10.1186/s13014-022-02043-0)
Supplement: Supplementary file 1 — Additional file 1. Example of a mapping table to map ROI names to variable names. CT-scan, computed tomography scan; Gy, Gray; SIB, simultaneous integrated boost In order to let departments use their own ROIs names they had to fill out a mapping table. a. ROIs descriptions mentioned in the table are examples and may differ per institution b. Based on the average dose calculated in the ROIs and if within certain ranges, mapped to prescribed dose c. Based on total dose in the boost PTV (SIB: elective + boost dose; sequential: boost dose). [file 13014_2022_2043_MOESM1_ESM.docx]

**Additional file 1**

Additional file 1: Example of a mapping table to map ROI names to variable names

| **Variable** | **Values** | **ROI description examples^a^** |
| --- | --- | --- |
| Laterality | Left vs right | Based on location centre of mass PTV relative to CT-scan centre |
| Which target areas are irradiated? *(Based on specific ROI name and whether it contains contours or not)* | Whole breast vs partial breast, chest wall | CTVp1^Breast vs CTVp1^PBI vs CTVp1^ThoracicWall |
|  | Boost breast or chest wall vs lymph nodes | CTVp1_Boost, CTVp_boost |
|  | Interpectoral | CTVn_Interpectoralis |
|  | Axillary lymph node levels I-II | CTVn_L1+L2+Interpectoralis |
|  | Axillary lymph node levels III-IV | CTVn_L1-L4 or CTVn_L3 and/or CTVn_L4 |
|  | Internal mammary lymphnodes | CTVn_IMN |
| Radiotherapy dose (local/tumour bed/regional) | Number of fractions | DICOM tags |
|  | Dose per fraction (Gy)^b^ | Mean dose mapped to standard fractionation |
|  | PTV dose homogeneity | DVH points from several ROIs |
|  | OAR dose | DVH points from several ROIs |
| Type of boost^c^ | SIB vs sequential boost | SIB if a plan was found with a total dose elective + boost;  sequential boost if a plan was found with only boost dose |

CT-scan, computed tomography scan; Gy, Gray; SIB, simultaneous integrated boost

In order to let departments use their own ROIs names they had to fill out a mapping table.

1. ROIs descriptions mentioned in the table are examples and may differ per institution
2. Based on the average dose calculated in the ROIs and if within certain ranges, mapped to prescribed dose
3. Based on total dose in the boost PTV (SIB: elective + boost dose; sequential: boost dose)
